# Does SIGLEC8 localize to the subcellular compartment like the Alzheimer's disease protective CD33 splice variant?

**DOI:** 10.3389/fncel.2023.1124150

**Published:** 2023-04-13

**Authors:** Chirag Dhar

**Affiliations:** InterVenn Biosciences, South San Francisco, CA, United States

**Keywords:** SIGLEC, Alzheheimer's disease, CD33, SIGLEC8, sialic acid

Recent studies have shown that SIGLEC8, a member of the CD33-related Siglec receptor family, is highly expressed in brain microglia (Olah et al., 2018; Estus et al., 2019; Gonzalez-Gil et al., 2022). A recent study by Gonzalez-Gil et al. demonstrated that SIGLEC8 binds to large proteoglycans, including the receptor protein tyrosine phosphatase RPTPζ carrying *O*-linked terminally sialylated keratan sulfate chains. These chains are produced by the sialyltransferase ST3GAL4 and the keratan sulfate sulfotransferase CHST1 (Gonzalez-Gil et al., 2022). By examining the *Human Central Nervous System Cell Type Expression Correlates* website (Kelley et al., 2018), I confirmed SIGLEC8 expression in microglia and at lower levels in oligodendrocytes (Figure 1A). I was also able to ask where SIGLEC8 was likely to signal by looking at co-expression of the receptor, ligand and glycosylation enzymes in the brain. While the highest level of SIGLEC8 transcript was seen in the frontal cortex, it was also detected in the parietal cortex and the cerebellum. PTPRZ1 was expressed in multiple cortical lobes and in the cerebellum, the sialyltransferase ST3GAL4 followed a similar expression pattern, and CHST1 expression was observed in both the frontal cortex and cerebellum (Figures 1B, C). These data when taken together, suggest SIGLEC8 may primarily signal by interacting with RPTPζ decorated with terminally sialylated keratan sulfate on cerebellar astrocytes.

**Figure 1:**
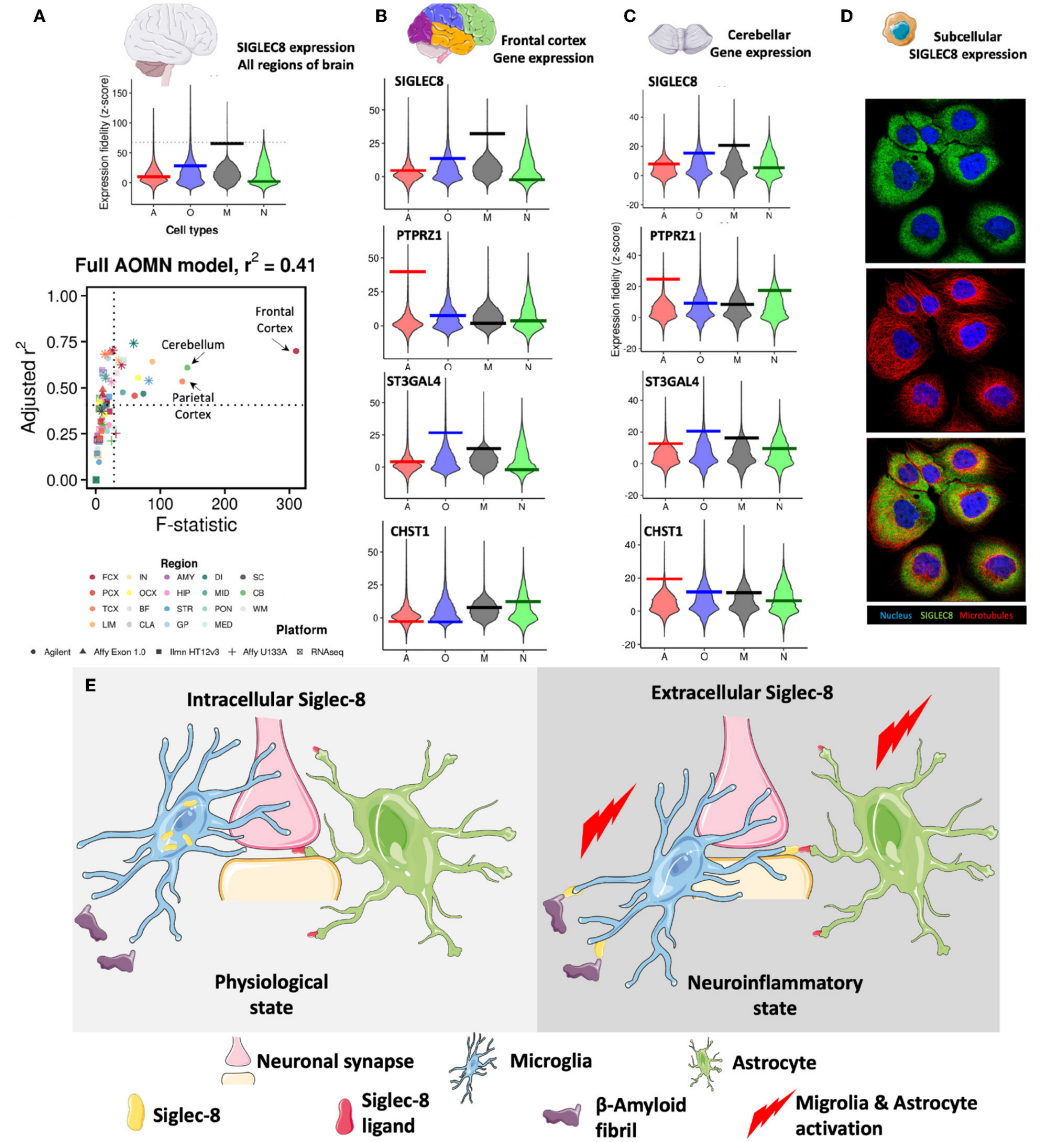
**(A)** SIGLEC8 is expressed by microglia and its expression is highest in the frontal cortex, parietal cortex, and the cerebellum. Genome-wide distributions of expression fidelity for astrocytes **(A)**, oligodendrocytes (O), microglia (M), and neurons (N) over all analyzed samples are shown. The horizontal line denotes the expression fidelity of the query gene for each cell type. The dashed horizontal line denotes the threshold above which all fidelity scores had Confidence = 100. Lower panel shows modeling results for expression levels of the SIGLEC8 as a function of variation in the inferred abundance of individual cell types. Simple linear regression is used to predict gene expression levels as a function of estimated neuron, astrocyte, oligodendrocyte, or microglia abundance in all human CNS regional datasets containing the query gene. Adjusted *R*^2^ values and t-values are shown for each cell type (labeled by color, with shapes denoting technology platforms). **(B)** Expression data of SIGLEC8, PTPRZ1, ST3GAL4, and CHST1 in the frontal cortex. **(C)** Expression data of SIGLEC8, PTPRZ1, ST3GAL4, and CHST1 in the cerebellum [data for **(A–C)** obtained from (http://oldhamlab.ctec.ucsf.edu)] **(D)** SIGLEC8 is localized to the subcellular compartment. Immunofluorescence images of the A-431 cell line stained with the HPA012556 SIGLEC8 antibody (https://www.proteinatlas.org/ENSG00000105366-SIGLEC8/subcellular#img, Human Protein Atlas https://www.proteinatlas.org) **(E)** Hypothetical effects of intracellular and extracellular expression of SIGLEC-8 Region FCX, frontal cortex; PCX, parietal cortex; TCX, temporal cortex; LIM, limbic cortex; IN, insular cortex; OCX, occipital cortex; BF, basal forebrain; CLA, claustrum; AMY, amygdala; HIP, hippocampus; STR, striatum; GP, globus pallidus; DI, diencephalon; MID, midbrain; PON, pons; MED, medulla; SC, spinal cord; CB, cerebellum; WM, white matter. Some representative images were obtained from smart.servier.com.

Surprisingly, when I investigated SIGLEC8 localization in the cell, I found evidence for intracellular expression (Figure 1D). While this observation warrants detailed molecular studies, it suggests that SIGLEC8 localization might be regulated in a similar fashion as the CD33 form (CD33m) that protects from Late Onset Alzheimer's Disease (LOAD) (Hollingworth et al., 2011) and it corroborates the hypothesis that Siglec receptors might exhibit intracellular functions. In fact, the evidence that the LOAD-protective *CD33* allele results in the expression of a protein unable to bind its natural ligand led to the suggestion that protection from disease was driven by a loss-of-function mechanism (Bradshaw et al., 2013; Griciuc et al., 2013). Parallel studies (Saha et al., 2019) have indicated that CD33m retention in intracellular peroxisomes prevented it from signaling (Siddiqui et al., 2017; Saha et al., 2019), adding credit to the loss-of-function hypothesis. However, the recent discovery of a non-functional CD33 allele not associated with LOAD has challenged this view.

There have been very few mechanistic studies exploring Siglec-8 in neurodegeneration. One that stands out mainly utilizes *in vivo* models of neurodegeneration utilizing Siglec-F—is the mouse paralog of Siglec-8 (Siddiqui et al., 2019)—and human cell line models to elucidate effects of Siglec-8 expression (Morshed et al., 2020). The investigators first identified a common response involving Siglec-F and Inpp5d likely linked to microglia (Morshed et al., 2020). They also explored RNASeq data and showed that aging-associated microglia had higher expression levels of Siglec-8 (Morshed et al., 2020). They also showed by immunofluorescence imaging, that Iba1+/MHC-II+ microglia in patients with LOAD had

the highest level of Siglec-8 expression (Morshed et al., 2020). Similar changes were noted in Siglec-F expression levels in mouse microglia during aging and neurodegeneration (Morshed et al., 2020). Additionally, they show Siglec-F and Siglec-8 induce cell death in a similar ITIM-dependent manner (Morshed et al., 2020). Lastly, the authors investigated the *in vitro* effects of IFNγ and TGFβ upregulate the expression of Siglec-8 in induced pluripotent stem cell-derived microglia (Morshed et al., 2020). The authors go on to suggest that targeting pathways downstream of Siglec-8/F may reduce neuroinflammation due to chronic receptor activation (Morshed et al., 2020).

It must be noted here that several variants of SIGLEC8 have been identified, and some of these (rs36498, rs10409962, and rs11672925) have been linked to asthma (Gao et al., 2010; Angata, 2014). It remains to be seen what the effect of these variants are on microglial Siglec-8 expression and function and if these are similar to the CD33 variants. Taking all these data together, I opine that intracellular Siglec receptors might instead have distinct roles from cell surface Siglecs, and they might modulate cell activation or protect from disease. Briefly, I suggest two distinct states of Siglec-8 expression exist (Figure 1E). One is an intercellular pool that does not interact with either sialylated β-amyloid proteins/prions (Srivastava et al., 2015) or astrocytes displaying sialylated keratin sulfate chains. These promote normal microglial phagocytic function and astocyte quiescence. On the other hand, Siglec-8 when expressed on the surface of microglia it binds to its ligands on the β-amyloid proteins and astrocytes leading to activation of these cell types, leading down neurodegenerative pathways.

Of course, it may be that Siglec-8 expression is simply modulated by aging processes, and not genetics. In this opinion piece, I also did not consider Siglec-8's other important ligand-−6'-sulfo sialyl Lewis X (O'Sullivan et al., 2018)—and its expression in the brain. It may be that these interactions are all working in concert in promoting neurodegenerative processes! These unaddressed issues are likely to be better understood when additional mechanistic experiments of Siglec-8 and its role in the brain are carried out. Future studies that address the potential roles of Siglecs retained within the cell will be particularly useful in understanding Alzheimer's disease and other cerebellar disorders like age-related cerebellar atrophy.

## Author contributions

The author confirms being the sole contributor of this work and has approved it for publication.
